# Genome-Wide Analysis of Milk Production Traits and Selection Signatures in Serbian Holstein-Friesian Cattle

**DOI:** 10.3390/ani14050669

**Published:** 2024-02-21

**Authors:** Marko Ristanic, Minja Zorc, Uros Glavinic, Jevrosima Stevanovic, Jovan Blagojevic, Milan Maletic, Zoran Stanimirovic

**Affiliations:** 1 Department of Biology, Faculty of Veterinary Medicine, University of Belgrade, Bul. Oslobodjenja 18, 11000 Belgrade, Serbia; mristanic@vet.bg.ac.rs (M.R.); rocky@vet.bg.ac.rs (J.S.); jovan.blagojevic@vet.bg.ac.rs (J.B.); zoran@vet.bg.ac.rs (Z.S.); 2 Department of Animal Science, Biotechnical Faculty, University of Ljubljana, Groblje 3, 1000 Ljubljana, Slovenia; minja.zorc@bf.uni-lj.si; 3 Department of Reproduction, Fertility and Artificial Insemination, Faculty of Veterinary Medicine, University of Belgrade, Bul. Oslobodjenja 18, 11000 Belgrade, Serbia; maletic@vet.bg.ac.rs

**Keywords:** GWAS, ROH, F_ROH_, inbreeding, milk production, cattle, Holstein-Friesian, milk proteins

## Abstract

**Simple Summary:**

This study focuses on identifying the genetic factors associated with milk-related traits in dairy cows. This research employed various genomic techniques to discover a significant association between a specific gene marker and milk protein concentration. The study also assessed inbreeding levels and revealed insights into the genetic mapping of dairy cows. We identified a marker that is significantly associated with milk protein concentration in first lactation (adjusted to 305 days) and, in addition to this marker, we also revealed genomic regions under selection pressure for other economically important traits. Moreover, we revealed low inbreeding levels among the tested animals. These findings contribute to enhancing breeding programs for Holstein-Friesian cattle and improving milk production.

**Abstract:**

To improve the genomic evaluation of milk-related traits in Holstein-Friesian (HF) cattle it is essential to identify the associated candidate genes. Novel SNP-based analyses, such as the genetic mapping of inherited diseases, GWAS, and genomic selection, have led to a new era of research. The aim of this study was to analyze the association of each individual SNP in Serbian HF cattle with milk production traits and inbreeding levels. The SNP 60 K chip Axiom Bovine BovMDv3 was deployed for the genotyping of 334 HF cows. The obtained genomic results, together with the collected phenotypic data, were used for a GWAS. Moreover, the identification of ROH segments was performed and served for inbreeding coefficient evaluation and ROH island detection. Using a GWAS, a polymorphism, rs110619097 (located in the intron of the *CTNNA3* gene), was detected to be significantly (*p* < 0.01) associated with the milk protein concentration in the first lactation (adjusted to 305 days). The average genomic inbreeding value (F_ROH_) was 0.079. ROH islands were discovered in proximity to genes associated with milk production traits and genomic regions under selection pressure for other economically important traits of dairy cattle. The findings of this pilot study provide useful information for a better understanding of the genetic architecture of milk production traits in Serbian HF dairy cows and can be used to improve lactation performances in Serbian HF cattle breeding programs.

## 1. Introduction

The Holstein-Friesian (HF) is the leading dairy breed in the world; its genetic potential for high milk yields is being exploited in virtually all developed countries. Milk production and milk composition are the most carefully selected traits in dairy cattle breeding programs [1,2]. The discovery of molecular markers associated with the aforementioned traits and the application of marker-assisted selection (MAS) made a huge impact on dairy cattle breeding in recent decades. To improve the genomic evaluation of milk-related traits and to comprehend the underlined molecular mechanisms, it is essential to identify the trait-associated genomic areas and candidate genes [3]. When introduced, genome-wide association studies (GWASs), took the lead over traditional QTL methods both in power of detecting causative gene variants and in defining narrower genomic regions containing causal variants [4]. Since GWASs are ideal for discovering genes that encode complex traits and their mechanisms [5], they have been widely conducted in recent years to identify relationships between genomic variants and economically significant traits in dairy cattle populations. As a result, a large number of candidate genes and quantitative genomic areas were detected to be associated with economically important traits, including milk-related traits, fertility, growth, and meat and carcass quality [3,4,6,7,8,9,10,11,12,13,14,15,16,17]. GWASs conducted in various cattle breeds utilized different SNP chip platforms and were focused on the identification of potential candidate genes and genomic regions responsible for milk production traits including milk yield (MY), fat yield (FY), and protein yield (PY). The most frequently reported candidate genes (DGAT1, GHR, MAPK15) and SNPs associated with milk production traits are located on chromosome 14 [4]. Other genes of interest harbored on chromosomes 14 and 20 are GNA14, PTBP2, and U6 which were closely associated with a higher MY [6,12,18], respectively. ITPR2 [19], ABCC9 [20,21], CPSF1 [21], PDE4 [13], and METTL15 [19] genes were reported to influence the expression of FY, while the genes associated with PY were PDHA2 [19], CTBP2 [19], MAPK9 [22], HPS3 [23], ARFGEF [24], SLCO1A2 [21], and MFSD1 [13].

The availability of a large number of known single nucleotide polymorphisms (SNP) contributed to the research regarding genomic selection and genomic inbreeding [25,26,27,28]. Genomic selection based on SNP chip genotypes relies on linkage disequilibrium (LD) between the QTL and the SNPs [29]. Traditionally, inbreeding was calculated by estimating inbreeding coefficients based on the pedigrees. The progress of modern genotyping methods enabled the increased use of genomic information for more precise estimation of inbreeding coefficients [30]. Several studies have shown that the characterization of inbreeding based on long runs of homozygous (ROH) genotypes presents a more accurate method of measuring individual autozygosity compared to the estimation of total inbreeding based on pedigree data [31,32,33]. The accuracy of the pedigree-based estimation of the inbreeding coefficient depends on the quality and completeness of the pedigrees. Pedigrees provide information on the expected proportion of the genome that is identical by descent (IBD), while the quantification of realized IBD can be measured directly with molecular markers. Furthermore, the genomic inbreeding coefficient provides an assessment of inbreeding by measuring the actual homozygosity. Moreover, the analysis of runs of homozygosity (ROH) provides insights into historical inbreeding patterns within the population and reveals genomic regions with inbreeding effects. Analyses of ROH regions can be used for animal breeding plans in order to minimize the adverse effects of inbreeding. Several studies have investigated the association of ROH with unfavorable gene variants in farm animals [34,35,36]. The signatures of natural and artificial selection “imprinted” on the genome can be traced back and contribute to a better understanding of the evolutionary processes that shaped the cattle genome [37,38,39]. The identification of genes and genomic regions affected by selection is essential to understand the biological mechanisms underlying the phenotypic differences observed between different species of farm animals that have different purposes and are influenced by different environmental conditions [37].

To the best of our knowledge, there is no official HF breeding program in Serbia, and in past decades, we were dependent on “genetics from abroad”, which is one of the reasons why we conducted this study. Furthermore, SNP chip analyses have never been performed before on Serbian HF dairy cattle, which is why this presents a very significant pilot study. Therefore, we decided to perform a GWAS using different software to analyze the association between SNPs and important production traits in a small population of Serbian HF cattle. In addition, the association between SNPs and inbreeding was assessed.

## 2. Material and Methods

### 2.1. Sampling

The material used for DNA extraction and further molecular-genetic analyses was collected from 334 chosen cows of the HF breed (that just finished their second lactation) from the farm units of the Al Dahra Corporation, Belgrade, Serbia (44.928635, 20.438109), the largest farm in Serbia, with approximately 4000 lactating cows. All animals were in good condition, without significant health problems within the first two lactations, and all were kept under the same conditions, in a tied system and received the same feed. All of these were criteria for inclusion in the study.

Blood was collected aseptically from the coccygeal vein in 10-mL vacutainers with added anticoagulant (EDTA) and stored at 4 °C until DNA extraction. All blood samples were collected during regular activities related to the implementation of animal health measures. New sterile gloves, needles, and vacutainers were used for each sample collection. All cows were kept under the same conditions, in a tied farm system and fed the same diet.

A total of 9289 AT4 milk records were taken from the database of the farm units of Al Dahra Corporation. Phenotypic data include total milk yield, milk protein, milk fat concentration, as well as milk yield, milk protein and milk fat concentration adjusted to 305 days for 334 genotyped HF cows. Cows were in their first to second parity. Analyzed traits were: trait 1: total milk yield in lactation 1, trait 2: total milk yield in lactation 2, trait 3: total milk protein concentration in lactation 1, trait 4: total milk protein concentration in lactation 2, trait 5: total milk fat concentration in lactation 1, trait 6: total milk fat concentration in lactation 2, trait 7: milk yield adjusted to 305 days in lactation 1, trait 8: milk yield adjusted to 305 days in lactation 2, trait 9: milk protein concentration adjusted to 305 days in lactation 1, trait 10: milk protein concentration adjusted to 305 days in lactation 2, trait 11: milk fat concentration adjusted to 305 days in lactation 1, trait 12: milk fat concentration adjusted to 305 days in lactation 2.

### 2.2. DNA Extraction and Genotyping

DNA extraction was performed using a commercial kit for BLOOD Version II (Lucigen Corporation, St. Middleton, WI, USA) according to the protocol of the Animal Production and Health Laboratory, International Atomic Energy Agency—IAEA, Seibersdorf, Vienna, Austria, which was additionally adapted for the purposes of this study. Briefly, the extraction was conducted from 1 mL of whole blood and consisted of removal of red blood cells, lysis of white blood cells, and precipitation and pelleting of the genomic DNA. The extracted DNA samples were quantified using a UV-VIS spectrophotometer (BioSpec-nano, Shimadzu Scientific Instruments, Kyoto, Japan) in order to check the DNA quality.

After DNA extraction, we performed genotyping of the extracted samples using the SNP chip Axiom Bovine BovMDv3 (ThermoFisher Scientific, Waltham, MA, USA) with 63,648 markers in collaboration with the IAEA and their laboratory (Animal Production and Health Laboratory, IAEA, Seibersdorf, Vienna, Austria). The analyses were performed according to the established protocol of the above-mentioned laboratory.

### 2.3. Quality Control of SNP Chip Data

Quality control of SNP chip data was performed using SNP & Variation Suite v8.9.1 software (Golden Helix, Inc., Bozeman, MT, USA). Samples with a genotyping success rate ≤ 0.90 were removed. In addition, markers were removed if the genotyping success rate was <0.95, if they had >2 alleles, or if the minor allele frequency (MAF) was <0.01. The data were then filtered to remove markers that were in linkage disequilibrium (LD), while leaving certain markers to represent groups that were linked to each other. This procedure left 29,503 out of 63,648 markers in 307 animals. MAF and LD purification was skipped to analyze the ROH segments, leaving 52,934 markers in 307 animals. In the data set for the ROH analyses (52,934 markers), we did not include markers from sex chromosomes, while in the GWAS analysis (29,503 markers), we included markers from the X chromosome in addition to the markers from autosomes.

### 2.4. GWAS

A GWAS was performed using the GAPIT3 library for R [40] with GLM (general linear model), MLM (multiple loci mixed model), FarmCPU (fixed and random model circulating probability unification), Super (settlement of MLM under progressively exclusive relationship), ECMLM (enriched CMLM), CMLM (compressed MLM), and Blink (Bayesian information and linkage-disequilibrium iteratively nested keyway). We considered the population structure and genetic relations within the studied population by performing principal component analysis and calculating the genotype relatedness matrix (kinship). The kinship matrix and the first three principal components (PCs) were fitted as covariate variables in the association models. We used the Bonferroni adjustment to determine GWAS significance cut-off (α  =  0.01).

### 2.5. Identification of ROH Segments

We identified ROH segments using SNP & Variation Suite v8.9.1 software (Golden Helix, Inc., Bozeman, MT, USA). We defined ROH as 25 or more consecutive homozygous markers on a segment with a minimum length of 1000 kilo base pairs (Kbp). Heterozygous markers were not allowed and there were no more than five unread SNPs. ROHs were classified based on their length (1–2 Mbp, 2–4 Mbp, 4–8 Mbp, 8–16 Mbp and >16 Mbp).

### 2.6. Genomic Inbreeding Coefficient Based on ROH

The genomic inbreeding coefficient (F_ROH_) [41] was calculated for each animal as the proportion of autosomal genome in ROH. The autosomal genome of *Bos taurus* is set at 2,512,082,506 bp. We calculated (F_ROH_) including all classes of ROH segments (1–2 Mbp, 2–4 Mbp, 4–8 Mbp, 8–16 Mbp and >16 Mbp). By adding the ROH segments of each class, we also calculated F_ROH1–2 Mb_, F_ROH2–4 Mb_, F_ROH4–8 Mb_, F_ROH8–16 Mb_ and F_ROH > 16 Mb_.

### 2.7. Detection of ROH Islands

We estimated the frequency of SNPs in ROH (%) and then plotted them against their positions along the chromosomes (Manhattan plot) using the statistical software library for R [42]. The minimum threshold for ROH island detection was set at 30%, meaning that ROH must be present in at least 30% of the population for a locus to be included in an ROH island [43].

## 3. Results

### 3.1. Genome-Wide Association Study—GWAS

**Figure 1:**
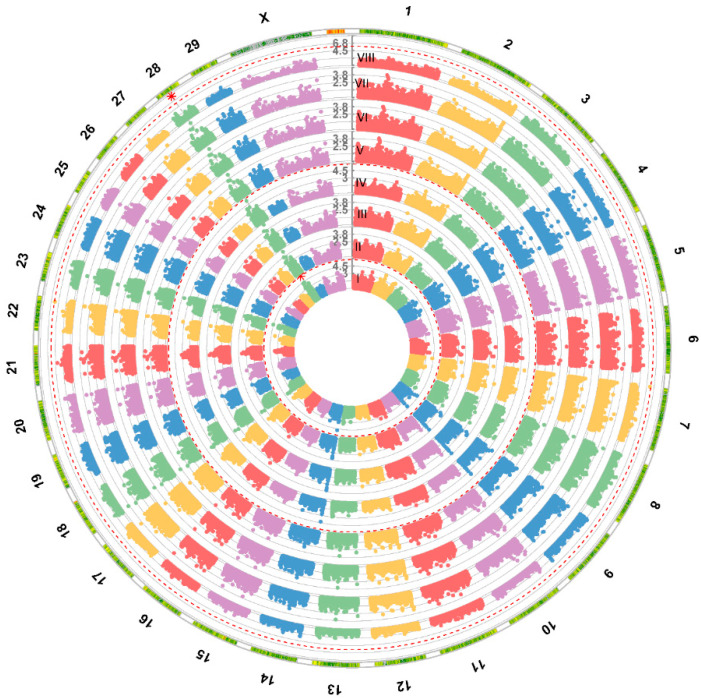
The circular Manhattan plot shows the results of eight different models: (I) GLM, (II) MLM, (III) MLMM, (IV) FarmCPU, (V) Super, (VI) ECMLM, (VII) CMLM, and (VIII) Blink; GWAS analysis of milk protein concentration (adjusted to 305 days) in the first lactation. The overlap in results between two different models is shown as a dashed vertical line extending through the Manhattan plots for all models used.

**Figure 2:**
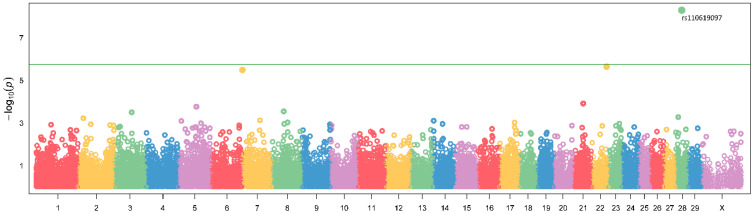
Manhattan plot of the GWAS analysis for the milk protein concentration (adjusted to 305 days) in the first lactation. The plot represents the −log_10_ transformed *p* values for all analyzed SNPs, green line presents GWAS significance level.

**Figure 3:**
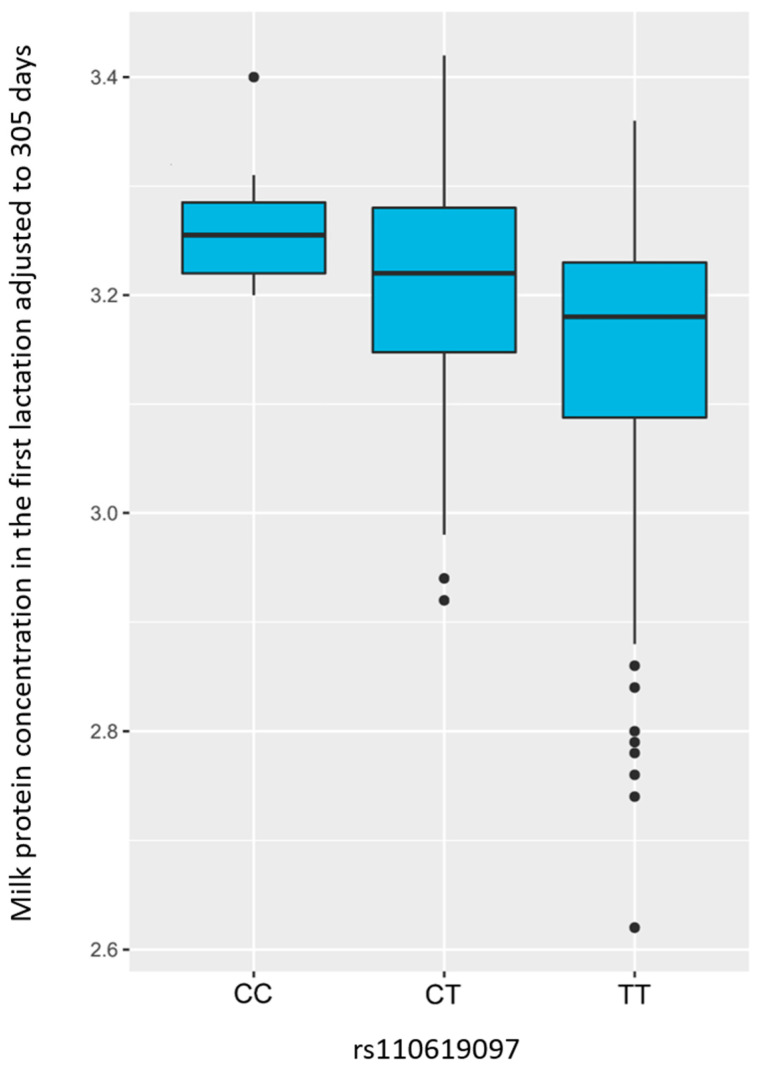
Milk protein concentration (adjusted to 305 days) in the first lactation with three different genotypes in the rs110619097 (28:g. 22479232T> C). X axis: different genotypes; Y axis: milk protein concentration.

The genome wide association study was performed for milk production traits: (milk yield, milk protein concentration, and milk fat concentration) using eight different models (GLM, MLM, MLMM, FarmCPU, Super, ECMLM, CMLM, and Blink). The GWAS revealed an association of SNP rs110619097 located on BTA28 using GLM and Blink models with protein concentration (adjusted to 305 days) in the first lactation (Figure 1). We examined the Q–Q plots generated by the different models; Blink generated the line close to a 1:1 ratio with an upward deviated tail (Supplementary Figure S1). The SNP rs110619097 (28:g. 22479232T>C, UMD3.1.1) significantly (*p* < 0.01) associated with milk protein yield (adjusted to 305 days) is located on the BTA28 chromosome (Figure 2). The MAF for this SNP is 0.18 (*p* < 0.01). This marker is located within the intron of the *CTNNA3* gene. The values of the milk protein concentration (adjusted to 305 days) in the first lactation for animals with different genotypes of the SNP rs110619097 were: CC: 3.263 ± 0.060 (10 animals), CT: 3.204 ± 0.109 (88 animals) and TT: 3.144 ± 0.134 (208 animals), undefined genotype: 3.320 (1 animal) (Figure 3). No other significant association of markers with investigated milk production traits (milk yield and milk fat concentration) was observed.

### 3.2. Runs of Homozygosity—ROH

**Figure 4:**
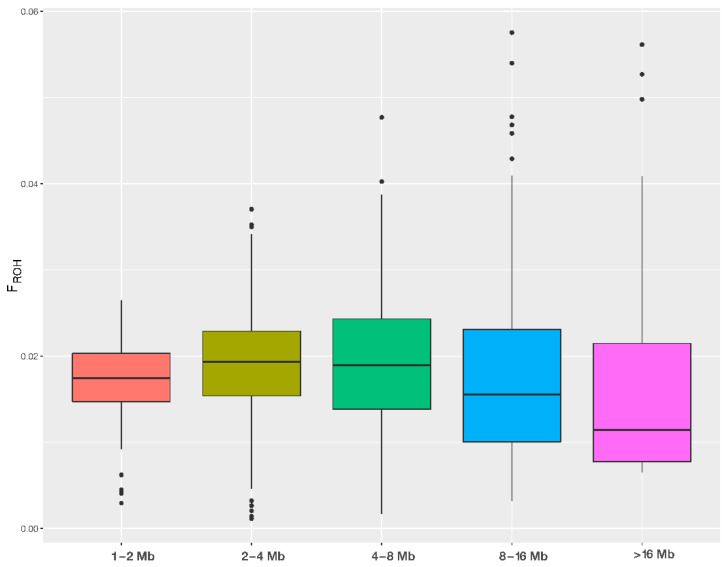
Boxplot distribution of genomic inbreeding (F_ROH_) obtained from different ROH length categories.

After refining the SNP chip data, we used 52,934 markers to identify ROH segments in the genomes of the studied HF cows. We identified a total of 18,549 ROHs with 60.42 ROHs per animal ranging from 8 to 95. We classified ROHs into five different categories based on their length: 1–2 Mbp, 2–4 Mbp, 4–8 Mbp, 8–16 and >16 Mbps. Descriptive statistics for each length category are shown in Table 1. The majority of the detected ROHs were in the 1–2 Mbp length category (Table 1). The longest ROHs (>16 Mbp) were the least, with no ROHs in this category observed in 45% of animals (*n* = 139). The average length of the identified ROHs is 3.28 Mbp. The longest identified ROH is 65.31 Mbp long and is located on chromosome BTA7. The total length of all ROH segments per animal is 197.92 Mbp, where in the animal with the largest total ROH length (433.37 Mbp), 17% of the autosome is covered by an ROH. The value of genomic inbreeding (F_ROH_) in that animal is 0.173, and in the least inbred animal, it is 0.005. Genomic inbreeding in the studied population is on average 0.079. For each length category, the inbreeding coefficient was calculated as the proportion of the autosomes of each individual that were covered by an ROH from that class (e.g., F_ROH_ > 16) (Figure 4).

### 3.3. Selection Signatures

**Figure 5:**
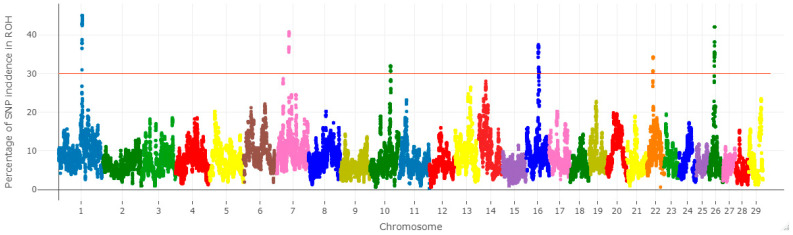
Manhattan plot distribution of ROH islands on chromosomes 1, 7, 10, 16, 22, and 26. The X-axis represents the chromosomal distribution of ROHs across the genome; The Y-axis shows the frequency of overlapping ROHs that are common in the tested samples.

In order to examine the effect of selection in the population of HF cows, we investigated the occurrence of ROH segments in the genomes of the examined individuals. The ROH incidence was not uniform across chromosomes (Figure 5). We identified ROH islands on chromosomes 1, 7, 10, 16, 22, and 26 (Supplementary Table S1). The highest ROH rate was observed on chromosome 1 (45% of animals) on markers AX-106742409 (rs41578805), AX-106740136 (rs109562914), AX-106719581 (rs41603780), AX-185115133 (rs523828967), and AX-124379394 (rs110792335).

## 4. Discussion

Various methods are being used to make breeding and selection programs more efficient [44]. Milk is a very important source of proteins and microelements and remains one of the most important components of the infant and adult diet. Dairy is a major dietary component of the human diet [45,46]. Having this in mind, the need for greater genetics of high-lactating cows is bigger than ever. Our study contributes to a better understanding of the genetics which underlie the milk productivity traits of cattle.

Even though GWASs have been conducted in cattle extensively in recent years, the available data are still insufficient to provide a complete explanation of the genetic mechanisms for milk production traits. As a result of numerous studies, hundreds of thousands of QTLs, which are responsible for the 680 traits [47], have been discovered on 30 chromosomes of cattle, and the list is still expanding. In our study, 334 HF cows with corresponding phenotypic data on milk yield, milk protein, and milk fat concentrations were genotyped and included in the GWAS. In this first of a kind study in Serbia, we found a significant correlation between the *CTNNA3* gene on the BTA28 chromosome and milk protein concentrations in the first lactation (adjusted to 305 days). In the study conducted on American HF cows, Cole et al. [18], similarly to our investigation, discovered a significant association between the *CTNNA3* gene and milk protein content (%), milk protein yield, milk fat content (%), milk fat yield, and milk yield. Using the GenABEL library for R software, v. 4.3.2, Dadousis et al. [48] observed the association of the same gene with other parameters that are important for dairy cattle (%CYSOLIDS, RECSOLIDS, and RECenergy—parameters related to curd dry matter). In general, many statistical methods and software packages are used for GWASs to improve computer efficiency, the power of statistical processing, and control of false positive results. In our research, we used GAPIT Version 3 software, which provided us with the possibility of comparing the results of association analyses using various statistical models (GLM, MLM, MLMM, FarmCPU, Super, ECMLM, CMLM, and Blink). In our study, the *CTNNA3* gene exerted an association with milk protein concentration when each of these statistical models was applied, indicating the potential of this marker in future dairy cattle breeding and selection programs. It is interesting to mention that we did not observe any significant signals from some of the most commonly discussed genes (e.g., DGAT, CSN). This could be due to the specificity of our study, such as a relatively small number of samples obtained from only one farm.

The level of inbreeding in a population is an important parameter for monitoring its genetic diversity and its management. High levels of inbreeding cause inbreeding depression and should be avoided in farm animals [49]. Moreover, increasing inbreeding is associated with negative effects on the production and reproductive characteristics of dairy cows [50,51]. Inbreeding is traditionally measured using pedigree data. However, the pedigree-based inbreeding coefficient has certain limitations. Inbreeding is a widely accepted concept for characterizing the evolution, diversity, and general structure of a population, and it can be studied at different levels, starting from the individual, herd, and up to the population level [48,52] or even within a consortium, such as an artificial insemination center or dairy farm [53]. Furthermore, controlling the degree of inbreeding in cattle populations is particularly important, as only a small number of animals from the entire population are used for breeding. This knowledge allows for better management of animal diversity and genetics, controlling inbreeding depression and, in smaller populations, it can be used for conservation purposes. To calculate genomic inbreeding in our study, we used genomic information obtained by means of a medium-density SNP chip (63,648 markers). With the SNP data that are obtained, the expected inbreeding values are replaced by the resulting homozygosity measurements, which are thought to be a more accurate way to assess inbreeding and better reflect the level of homozygosity. In our study, the least detected ROHs were the longest (>16 Mbp—indicating recent inbreeding), and 45% of the animals had no ROH > 16 Mb at all, which is similar to the results obtained by Doekes et al. [54], who in their research did not observe ROH > 16 Mb in 26% of the examined cows. Our findings are in line with previous studies [41,55,56] in which short ROHs (<2 Mbp) were observed more frequently than long ROHs. Our results of mean ROH counts per animal, observed across all categories (1–2 Mbp: 30.140; 2–4 Mbp: 17.505; 4–8 Mbp: 8.679; 8–16 Mbp: 3.992; >16 Mbp: 1.726, respectively), are in accordance with the results of Marras et al. [55], who determined the following values: 1–2 Mbp: 46.5; 2–4 Mbps: 17.0; 4–8 Mbps: 9.7; 8–16 Mbps: 5.9; >16 Mbps: 3.0. In our study, the average genomic inbreeding value (F_ROH_) was calculated using SVS Golden Helix software v8.9.0 and was 0.079, which is slightly lower than the values obtained by Marras et al. [55] in a population of Italian HF cattle (0.116), while Makanjuola et al. [57] obtained F_ROH_ values of 0.136 and 0.156, respectively, in a population of Canadian HF cattle, using SNP1101 version 1.0 and PLINK v1.90 software. Our results suggest the presence of a low level of recent and total inbreeding in the investigated population of Serbian HF cattle.

Identifying recent signatures of positive selection in domestic animals can provide information on the genomic regions affected by artificial and natural selection and thereby help identify beneficial mutations and underlying biological pathways for economically important traits [39]. There are several different approaches to detecting selection signatures, and we decided to utilize ROH analyses. ROHs are a state-of-the-art method for analyzing inbreeding in animal populations. Furthermore, ROHs are suitable for revealing selection signatures via ROH island identification. ROH islands can be defined as genomic regions with reduced genetic diversity and, consequently, high homozygosity around a selected locus that could contain targeted regions of positive selection, which are under strong selective pressure [43]. In our research, ROH islands were observed on chromosomes 1, 7, 10, 16, 22, and 26, while chromosome 1 had the highest rate of detected ROHs. ROH islands on chromosome BTA1 were also identified in other cattle populations by Purfield et al. [31], Mastrangelo et al. [58], and Gurgul et al. [59]. Our results indicate the existence of a 2 Mbp (starting at 83 Mbp and ending at 85 Mbp) ROH island on BTA1. In 45% of the animals from the studied population, selection signatures appear in the following genes: *PARL, YEATS2,* and *KLHL24*, while Serão et al. [60] observed an association of this region with residual feed intake (RFI) in the Angus and Simmental cattle breeds, which leads us to conclusion that the discovered ROH islands may be related to other productive/reproductive traits of cattle.

To summarize, by utilizing a GWAS, a significant association (*p* < 0.01) was observed between the rs110619097 polymorphism located in the intron of the *CTNNA3* gene of the BTA28 chromosome and milk protein concentration in the first lactation (adjusted to 305 days). The results of the ROH segment analyses revealed a low level of recent and total genomic inbreeding in the examined population of HF cattle. The detected ROH islands imply their link with the productive traits of dairy cows and present the regions of the genome that are under selection pressure for other economically important traits.

## 5. Conclusions

This study presents a pilot study in the field of the SNP genotyping of animals in Serbia. All of the obtained data regarding the GWAS and ROH analyses provide useful information for a better understanding of the genetic architecture of milk production traits and genomic inbreeding in dairy cows and present a good base for many more studies on Serbian HF cattle. Furthermore, we are keen to continue our research on larger number of Serbian HF cattle, expanding it to larger number of farms and looking for other important traits of dairy cows, such as health and reproductive traits.

## Figures and Tables

**Table 1:** ROH length, ROH number, and genomic inbreeding (F_ROH_) in different ROH length categories per animal.

		ROH Length Category				
	Parameter	1–2 Mbp	2–4 Mbp	4–8 Mbp	8–16 Mbp	>16 Mbp
Total length of ROH per animal	Average Value	43.848	48.119	48.145	44.038	39.332
	Standard Deviation	9.979	15.504	20.138	25.655	24.867
	Median	43.817	48.553	47.651	39.144	28.769
	Minimum	7.416	2.927	4.244	8.013	16.282
	Maximum	66.538	93.096	119.858	144.577	141.065
Number of ROH per animal	Average Value	30.140	17.505	8.679	3.992	1.726
	Standard Deviation	6.723	5.488	3.550	2.286	0.933
	Median	30.000	18.000	8.000	4.000	1.000
	Minimum	6.000	1.000	1.000	1.000	1.000
	Maximum	45.000	35.000	21.000	12.000	5.000
Genomic inbreeding F_ROH_	Average Value	0.017	0.019	0.019	0.018	0.016
	Standard Deviation	0.004	0.006	0.008	0.010	0.010
	Median	0.017	0.019	0.019	0.016	0.011
	Minimum	0.003	0.001	0.002	0.003	0.006
	Maximum	0.026	0.037	0.048	0.058	0.056

## Data Availability

The raw data supporting the conclusions of this article will be made available by the authors on request.

## Supplementary Materials

Please add: The following supporting information can be downloaded at: https://www.mdpi.com/article/10.3390/ani14050669/s1, Figure S1: Q-Q plot of GWAS for milk protein concentration in the first lactation (reduced to 305 days). Table S1: Detected selection signatures and the association found by other authors.
