# Supplementary material for: Genome-Wide Analysis of Milk Production Traits and Selection Signatures in Serbian Holstein-Friesian Cattle
Source: Animals (Basel). 2024 Feb 21;14(5):669. doi: 10.3390/ani14050669 (PMC10930642; doi:10.3390/ani14050669)
Supplement: Supplementary file 1 [file animals-14-00669-s001.zip › Supplementary_Ristanic_et_al_2023.pdf]

Supplementary Table S1. Detected selection signatures and the association found by other authors.

<table>
<tr><th>Chr.</th><th>Genomic location Mbp</th><th>Animals within ROH %</th><th>Genes</th><th>Previously published results</th></tr>
<tr><td rowspan="2">1</td><td rowspan="2">83-84</td><td rowspan="2">44.95</td><td rowspan="2">MAGEF1, MIR2284Y-3, EPHB3, LOC524771, THPO, POLR2H, CLCN2, FAM131A, PSMD2, <u>ECE2</u>, CAMK2N2, ALG3, MIR1224, ABCF3, AP2M1, HTR3C, ABCC5, PARL, MAP6D1</td><td>Identified signature of selection on the genomic region 1:82 820 000-84 650 000, in a local cattle population from North Africa (Ben-Jemaa et al. 2020)</td></tr>
<tr><td>The ECE2 gene was associated with susceptibility to respiratory diseases in Charolais, Hereford, Red Angus and Angus cattle (Neupane et al. 2018).</td></tr>
<tr><td>1</td><td>84-85</td><td>44.95</td><td>YEATS2, KLHL24, B3GNT5, LAMP3, MCCC1, DCUN1D1</td><td>Serão et al. (2013) found an association of this region with residual feed intake (RFI) in Angus and Simmental cattle breeds.</td></tr>
<tr><td>7</td><td>42-43</td><td>39.74</td><td><u>NLRP3</u></td><td>The NLRP3 gene is associated with resistance to infection with Mycobacterium avium ssp. paratuberculosis in HF cattle (Mallikarjunappa et al. 2018)</td></tr>
<tr><td>7</td><td>43-44</td><td>40.72</td><td>MGC137030, <u>LYPD8</u>, SH3BP5L, ZNF692</td><td>LYPD8 is involved in the biological processes of defense responses to gram-negative bacteria and is associated with adaptive responses to environmental stimuli such as stress, infection and inflammation in cattle (Poščić et al. 2017).</td></tr>
<tr><td>10</td><td>72-73</td><td>31.92</td><td>L3HYPDH, JKAMP, CCDC175, <u>DHRS7</u>, PPM1A, <u>SIX6</u></td><td>Gaddis et al. (2017) linked the DHRS7 and SIX6 genes to the number of embryos in HF cows.</td></tr>
<tr><td>10</td><td>73-74</td><td>31.92</td><td>MNAT1, TRMT5, <u>PRKCH</u></td><td>PRKCH was associated with back angle in HF cows (Yan et al. 2020).</td></tr>
<tr><td>16</td><td>42-43</td><td>36.81</td><td>DHRS3, VPS13D, <u>TNFRSF1B</u>, MIIP, <u>MFN2</u>, <u>PLOD1</u>, <u>KIAA2013</u>, <u>NPPB</u>, <u>NPPA</u>, <u>CLCN6</u>,</td><td>In the Yaroslav, Kolmogor and HF breeds, a selection signature was identified in the same region (Zinovieva et al. 2020).</td></tr>
</table>

			MTHFR, *MIR12050*, *AGTRAP*, *DRAXIN*, *MAD2L2*, *FBXO6*, *FBXO44*, *FBXO2*	*MTHFR* is involved in milk protein synthesis (Menzies et al. 2009).
16	43-44	37.46	*DISP3, ANGPTL7, EXOSC10, SRM, MASP2, TARDBP, PEX14*	*EXOSC10* is associated with longevity in HF cows (Steri et al. 2019).
16	44-45	36.81	*DFFA, CORT, CENPS, PGD, UBE4B, LOC112441838, NMNAT1, CTNNBIP1, PIK3CD, TMEM201*	*NMNAT1* is associated with reproductive efficiency of cows (Mota et al. 2017)
16	45-46	30.29	*SPSB1, MIR34A, SLC2A5, CA6, ENO1, MIR2285CK, SLC45A1, LOC786597*	*ENO1* gene was significantly associated with bull fertility (Park et al. 2012)
22	21-22	30.62	*EDEM1, ARL8B, BHLHE40, ITPR1, MIR2285AM*	*BHLHE40* is associated with milk protein content (Singh et al. 2022)
22	22-23	34.20	*SUMF1, SETMAR, LRRN1*	*SUMF1* gene is associated with milk yield in HF cows (Pedrosa et al. 2021)
26	21-22	37.13	*CHUK, CWF19L1, SCD, MIR12016, SEC31B, NDUFB8, HIF1AN, SLF2, SEMA4G, MRPL43, TWNK, LZTS2, SFXN3, TLX1, LBX1*	*SCD* gene is associated with medium- and long-chain unsaturated fatty acids in a population of Chinese HF cows (Li et al. 2016).
26	22-23	42.02	*BTRC, POLL, DPCD, FBXW4, FGF8, NPM3, OGA, KCNIP2, HPS6, PPRC1, NOLC1, ELOVL3, PITX3, GBF1, NFKB2, PSD, FBXL15, CUEDC2, MIR146B, MFSD13A, ACTR1A, SUFU*	Wang et al. (2017) observed a significant increase in *MIR146B* expression levels in subclinical, clinical and experimental mastitis-infected bovine mammary gland tissue.
26	23-24	34.85	*TRIM8, ARL3, SFXN2, MIR2392, CYP17A1, AS3MT, CNNM2, NT5C2*	*CNNM2* was associated with milk yield in HF cows (Jiang et al. 2019a).
