# Supplementary figures and images for: Genome-Wide Analysis of Milk Production Traits and Selection Signatures in Serbian Holstein-Friesian Cattle
Source: Animals (Basel). 2024 Feb 21;14(5):669. doi: 10.3390/ani14050669 (PMC10930642; doi:10.3390/ani14050669)

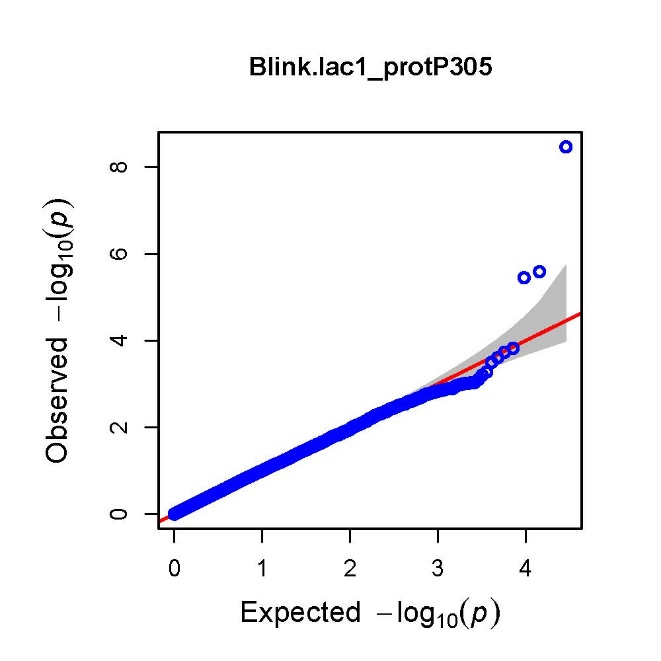

Supplement: Supplementary file 1 [file animals-14-00669-s001.zip › Supplementary Figure S1.tif]
